# A Fault Identification Method for Micro-Motors Using an Optimized CNN-Based JMD-GRM Approach

**DOI:** 10.3390/mi17010123

**Published:** 2026-01-19

**Authors:** Yufang Bai, Zhengyang Gu, Junsong Yu, Junli Chen

**Affiliations:** 1 College of Electrical Engineering, Shanghai Dianji University, Shanghai 201306, China; 19151762032@163.com; 2 Key Laboratory of Opto-Electronic Information Science and Technology of Jiangxi Province, Nanchang Hangkong University, Nanchang 330063, China; 18070283782@163.com; 3 College of Aeronautics, Shanghai Dianji University, Shanghai 201306, China; chenjl@sdju.edu.cn

**Keywords:** micro-motors, JMD method, global relationship matrix, optimized CNN

## Abstract

Micro-motors are widely used in industrial applications, which require effective fault diagnosis to maintain safe equipment operation. However, fault signals from micro-motors often exhibit weak signal strength and ambiguous features. To address these challenges, this study proposes a novel fault diagnosis method. Initially, the Jump plus AM-FM Mode Decomposition (JMD) technique was utilized to decompose the measured signals into amplitude-modulated–frequency-modulated (AM-FM) oscillation components and discontinuous (jump) components. The proposed process extracts valuable fault features and integrates them into a new time-domain signal, while also suppressing modal aliasing. Subsequently, a novel Global Relationship Matrix (GRM) is employed to transform one-dimensional signals into two-dimensional images, thereby enhancing the representation of fault features. These images are then input into an Optimized Convolutional Neural Network (OCNN) with an AdamW optimizer, which effectively reduces overfitting during training. Experimental results demonstrate that the proposed method achieves an average diagnostic accuracy rate of 99.0476% for multiple fault types, outperforming four comparative methods. This approach offers a reliable solution for quality inspection of micro-motors in a manufacturing environment.

## 1. Introduction

Micro-motors are essential power-drive components in industrial applications [1], distinguished by their high rotational speed, compact size, low weight, and high efficiency. These devices are widely used in military, medical, and aerospace sectors, among others [2]. Variations in equipment precision and inconsistencies in manufacturing and testing processes can result in faults such as commutator wear, shaft bending, and housing deformation [3]. These faults may introduce significant safety hazards, lead to economic losses, or result in personal injury. Consequently, the development of efficient and reliable fault diagnosis techniques is critical for accurately assessing the health status of micro-motors and identifying potential issues. Such advancements are vital for maintaining safe production and effective equipment maintenance.

Various methods are currently employed for signal detection in motor fault diagnosis, including vibration [4], current [5], and temperature [6] signals. Current signals are relatively easy to obtain and can be measured without direct contact; however, they may lack the sensitivity required to detect minor faults. Vibration signals are typically captured using large, heavy, fixed acceleration sensors, which are unsuitable for small micro-motors. In contrast, non-contact sensing technologies such as laser Doppler vibrometers [7], optical fibers [8], and acoustic sensors [9] provide high sensitivity, strong resistance to interference, and low power consumption. Due to the high cost of optical sensors, this study employs a non-contact, cost-effective acoustic sensor to collect the original signals.

The sound signal generated by the micro-motor is non-stationary, characterized by varying frequencies, amplitudes, and phases, as well as environmental noise and electromagnetic interference. Therefore, modal decomposition of the motor’s sound signal is necessary to extract information relevant to fault characteristics. Common signal processing techniques, including Wavelet Packet Decomposition (WPD) [10], Empirical Mode Decomposition (EMD) [11], and Variational Mode Decomposition (VMD) [12], are widely used for this purpose. Wavelet packet decomposition is most effective when an appropriate structure and basis functions are selected, but it is limited to stationary or periodic signals. In comparison, EMD [13] and VMD [14] are effective for analyzing oscillatory signals; however, both these methods are susceptible to mode aliasing in practical applications. Mojtaba Nazari et al. [15] introduced the Jump plus AM-FM Mode Decomposition (JMD) method to separate distinct signal components. This approach distinguishes between jump and oscillatory elements by reducing bandwidth and imposing penalties for signal discontinuity. The JMD method was initially applied to the analysis of the Earth’s electric field and medical signals, such as electrocardiograms (ECG) and electroencephalograms (EEG). In the context of motor faults, the resulting sound signals often contain jump components. The present study applies the JMD method to decompose and extract features from micro-motor sound signals, aiming to improve feature extraction accuracy.

Two-dimensional images provide a more effective means of conveying the dynamic features of sequences than one-dimensional signals, as they utilize texture and shape to emphasize these characteristics. Numerous researchers have developed diverse methods for signal visualization. For example, Liu et al. [16] introduced a model employing the Gram Angular Difference Field (GADF) and a self-calibrating convolution module, achieving classification accuracy exceeding 90%, which is 6% to 15% higher than other models. Yan et al. [17] applied the Markov Transition Field (MTF) in conjunction with a deep Residual Network (MTF-ResNet) to accurately identify both fault types and severity. Furthermore, Boudiaf et al. [18] integrated wavelet spectra derived from the Continuous Wavelet Transform (CWT) into a convolutional neural network (CNN), demonstrating superior accuracy and efficiency compared to traditional techniques on the American Mechanical Fault Testbed (MFPT) dataset. Unlike these approaches, the Global Relationship Matrix (GRM) effectively captures global relationships among signals without relying on specific state divisions or the selection of wavelet bases [19]. GRM enhances the contrast of graph textures relative to the relative relationship matrix, making it particularly suitable for detecting faults in weak signals. Therefore, GRM is employed as a feature extractor to obtain fault-sensitive features from micro-motor fault signals, which are subsequently used as inputs for machine learning algorithms in the fault diagnosis process.

With the advancement of machine learning, CNNs have become fundamental tools for image analysis and classification tasks [20]. Wang Y et al. [21] introduced a VMD-FFT feature extraction method integrated with a CNN-Transformer model, which achieved high diagnostic accuracy on the CWRU bearing dataset and demonstrated strong performance across multiple datasets. Ding H et al. [22] propose a parallel 2D Swin Transformer and 1D CNN architecture based on FFT-VMD feature extraction and enhanced by SE attention, improving accuracy and robustness for rolling bearing fault diagnosis.

Nevertheless, traditional CNNs face considerable challenges, including limited feature extraction, vanishing gradients, and tightly coupled tuning processes. To overcome these challenges, this study introduces an optimized CNN model. The LeakyReLU activation function is employed to alleviate the “neuron death” and enhance the representation of non-linear features [23]. Furthermore, we employ the AdamW optimizer [24] to effectively mitigate overfitting by decoupling the weight decay strategy, thereby stabilizing fault identification.

In conclusion, several critical issues necessitate further examination in the domain of micro-motor fault diagnosis:

(1) The acoustic signals obtained from micro-motors are highly susceptible to external interference, which complicates the extraction of meaningful signal features. Furthermore, traditional decomposition methods often exhibit mode aliasing;
(2) Traditional one-dimensional time series signals have limitations in feature ex-pression and it is difficult to visually present the time-dependent features and nonlinear features of these signals.
(3) Traditional CNNs still have shortcomings in fault image recognition, such as insufficient feature representation ability, a tendency to fall into vanishing gradients and overfitting. Consequently, achieving stable and efficient classification in fault image recognition under complex conditions remains a significant challenge.

To address these gaps, this study proposes a JMD–GRM–optimized CNN based micro-motor fault diagnosis framework. Unlike conventional decomposition methods that suffer from mode aliasing, JMD is first applied to explicitly extract AM–FM oscillatory components and discontinuous jump components, which are closely related to mechanical faults. GRM is then employed to convert the fused sequence into a two-dimensional grayscale map, effectively capturing global temporal correlations and highlighting weak fault features. Finally, an optimized CNN with AdamW decoupled weight decay is introduced to improve training stability and reduce overfitting. This paper is organized as follows: In Section 2, we conduct a comparative analysis of the signal decomposition accuracy between JMD and VMD, along with an assessment of the noise sensitivity of GRM. Furthermore, we develop a fault diagnosis model based on an optimized CNN using JMD-GRM. Section 3 presents a detailed description of the experimental platform and outlines the creation of a self-constructed dataset. In Section 4, we rigorously validate the proposed fault diagnosis method through a series of comparative tests, demonstrating its efficiency and accuracy. Finally, Section 5 summarizes the key conclusions of this study.

## 2. Basic Theory of JMD-GRM

### 2.1. The Principle of Jump Plus AM-FM Mode Decomposition

The signal measured during the operation of a motor is predominantly a complex mixture comprising oscillatory components, jumps, and periodic signals. While the traditional VMD method has shown excellent performance in extracting oscillatory components from data [25], its decomposition process depends on the specified number of modes and predetermined bandwidth constraints. Essentially, this method focuses on minimizing bandwidth in the frequency domain, which can lead to limitations when addressing signals that feature significant variations. The JMD approach addresses this issue by solving a variational optimization problem. It establishes a minimum bandwidth constraint on the oscillating component to ensure the concentration of spectral energy. In addition, sparse regularization is applied to the derivatives of the jumping component, enabling the capture of abrupt changes in the signal. This methodology allows JMD to effectively separate and delineate the frequency domains of the oscillating and jumping components.

Specifically, assuming that the motor signal is composed of three primary components: oscillation, jump, and noise. It is assumed that the signal follows a specific mathematical pattern [15,26]:

(1) $$f\left(t\right)=\overset{K}{\underset{k=1}{\sum }}u_{k}\left(t\right)+v\left(t\right)+n\left(t\right)$$

where $u_{k}\left(t\right)$ and $v\left(t\right)$ denote the oscillation and the jump component within the input data respectively, while $n\left(t\right)$ represents noise, K indicate the number of oscillatory components of the input signal.

(2) $$\begin{array}{l} \mathrm{Min}\left(J(\left\{u_{k}\right\},\left\{\omega_{k}\right\},v,x)\right)=\left\{\alpha \right.\cdot J_{1}\\ \left.+\beta \cdot J_{2}+{\left\|f\left(t\right)-\left(v\left(t\right)+\underset{k}{\sum }u_{k}\left(t\right)\right)\right\|}_{2}^{2}\right\}=\left\{\alpha \underset{J_{1}}{\underset{︸}{\underset{k}{\sum }{\left\|\partial_{t}\left[u_{k+}\left(t\right)e^{-j\omega_{k}t}\right]\right\|}_{2}^{2}}}\right.\\ \left.+\beta \underset{J_{2}}{\underset{︸}{\int_{0}^{\infty }\phi ({\left\|\partial_{t}v\left(t\right)\right\|}_{2}\;;\;b)\;dt}}+{\left\|f\left(t\right)-\left(v\left(t\right)+\underset{k}{\sum }u_{k}\left(t\right)\right)\right\|}_{2}^{2}\right\}\\ s.t.\;\;\;\;x=\partial_{t}v \end{array}$$

where $J_{1}$ represents the bandwidth of the AM-FM oscillation component, whereas $J_{2}$ represents a constraint function that incorporates a sparsity jump component; $\omega_{k}$ represents the central frequency of the *k*-th oscillation mode; α and β act as the weight parameter, serving to balance the oscillation component against the penalty strength of the jump component; $\partial_{t}$ denotes the time derivative. Additionally, ϕ(•:b) is defined as a piecewise function:

(3) $$\phi \left(x;b\right)=\left\{\begin{array}{c} -\frac{b}{2}x^{2}+\sqrt{2b}x\;\;x\in \left[0,\sqrt{2/b}\right)\\ 1\;\;\;\;\;\;\;\;\;\;\;x\in \left[\sqrt{2/b},+\infty \right) \end{array}\right.$$

**Figure 1:**
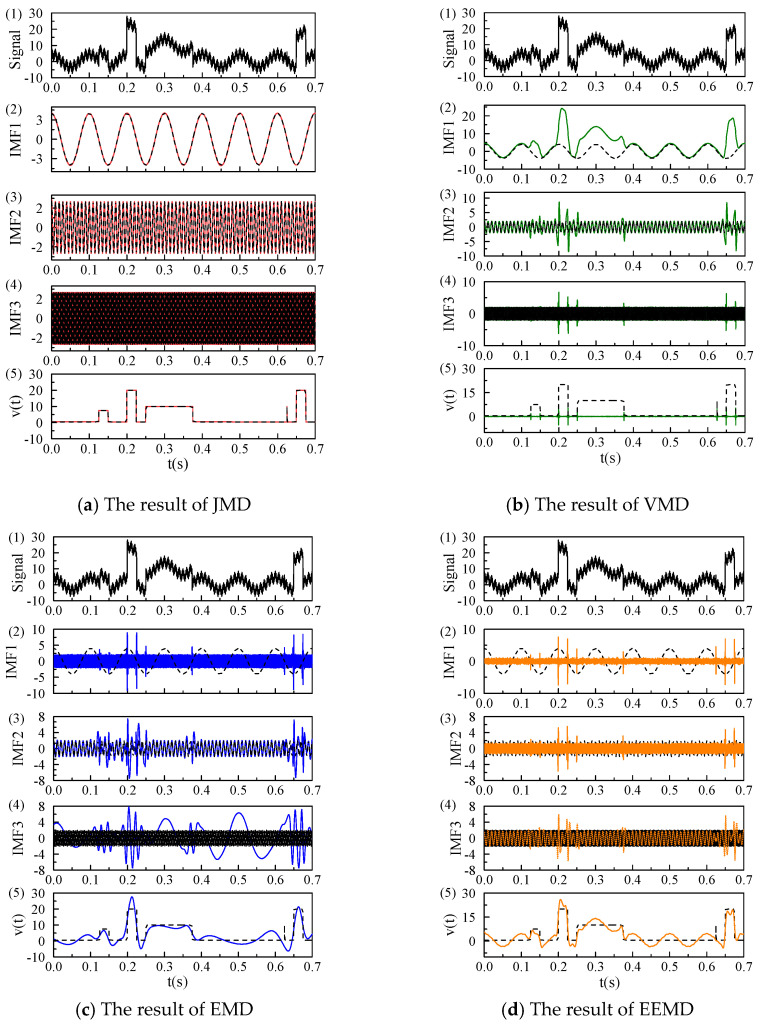
Comparison of JMD, VMD, EMD, and EEMD for the same signal, the dashed black lines represent the original components of the signal, while the red, green, blue, and orange solid lines represent the decomposition results of JMD, VMD, EMD, and EEMD, respectively.

In this section, we constructed a complex signal that consists of three sinusoidal components at frequencies of 10 Hz, 100 Hz, and 500 Hz, along with a jump signal and white Gaussian noise. We respectively adopted JMD, VMD, EMD, and Ensemble Empirical Mode Decomposition (EEMD) to analyze this signal to compare the accuracy of these four methods. The decomposition results are shown in Figure 1. A comparative analysis of subplots in (a), (b), (c), and (d) in Figure 1 indicates that the JMD method effectively separates the components corresponding to the three distinct oscillation frequencies and the jump component from the original signal. In contrast, the results obtained from VMD, EMD, and EEMD exhibit varying degrees of mode aliasing among the three oscillation modes.

**Figure 2:**
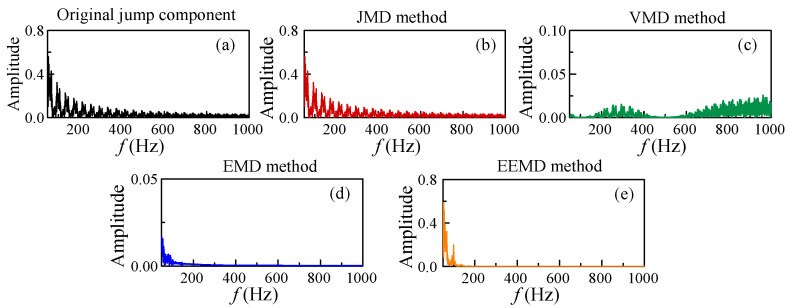
Comparison of the frequency spectra of the jump components obtained by (**b**) JMD, (**c**) VMD, (**d**) EMD, and (**e**) EEMD methods. Sub-figure (**a**) shows the frequency spectrum of the original jump section.

The limitations of VMD, EMD, and EEMD become apparent when analyzing the complex broadband spectral characteristics of jump components. To investigate this phenomenon, the spectral analysis was conducted on the jump signal after decomposition, as illustrated in Figure 2. Figure 2a–e display the spectra of the original jump signal and the jump decomposition variables derived from the JMD, VMD, EMD, and EEMD methods, respectively. The analysis results demonstrate that the JMD method effectively captures the entire jump signal, whereas the spectra generated by VMD, EMD, and EEMD are incomplete. Given the prevalence of jump signals in motor fault signals, JMD is a more suitable technique for motor fault detection compared to VMD, EMD, or EEMD methods.

### 2.2. The Global Relation Matrix (GRM) Method

Aiming at the problem that the time-domain characteristics of the sound signals of micro-motors under normal and fault conditions are not significantly different, this paper adopts the GRM method to convert the measured sound data into two-dimensional grayscale images [19]. First, the vibration signal $X=(x_{t},t=1,2,\ldots ,N)$ is dimensionally reduced and smoothed using the piecewise aggregation approximation (PAA) algorithm to obtain a time series $\tilde{X}=({\tilde{x}}_{1},\ldots ,{\tilde{x}}_{m})$ of length of m:

(4) $${\tilde{x}}_{i}=\frac{1}{r}\sum_{j=(i-1)r+1}^{ir}x_{j},i=1,2,\ldots ,m$$

Then the m×m global relation matrix M is constructed. In this matrix, the diagonal elements retain symbolic information, whereas the non-diagonal elements express the correlations, observed at distinct time points in the form of products:

(5) $$M_{ij}=\left\{\begin{array}{l} sign({\tilde{x}}_{i}),i=j\\ {\tilde{x}}_{i}\times {\tilde{x}}_{j},i\neq j \end{array}\right.$$

Finally, min-max normalization is performed on the matrix ***M*** to obtain the gray-scale matrix ***F***:

(6) $$F=\frac{M-min(M)}{max(M)-min(M)}\times 255$$

This methodology enhances global correlation across time points within the time series by facilitating interactions among matrix elements. It effectively captures nonlinear dynamic features of the signal, thereby offering comprehensive temporal texture information that is advantageous for subsequent feature extraction through optimized CNN.

**Figure 3:**
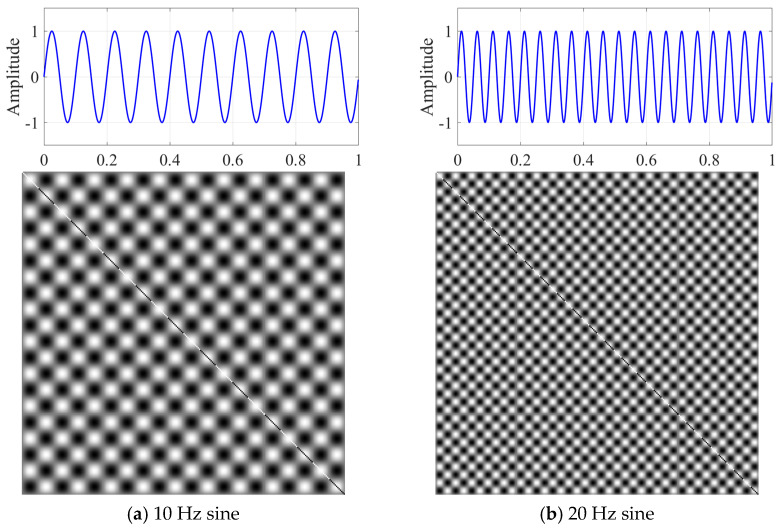
Sine time series data and images generated by GRM.

**Figure 4:**
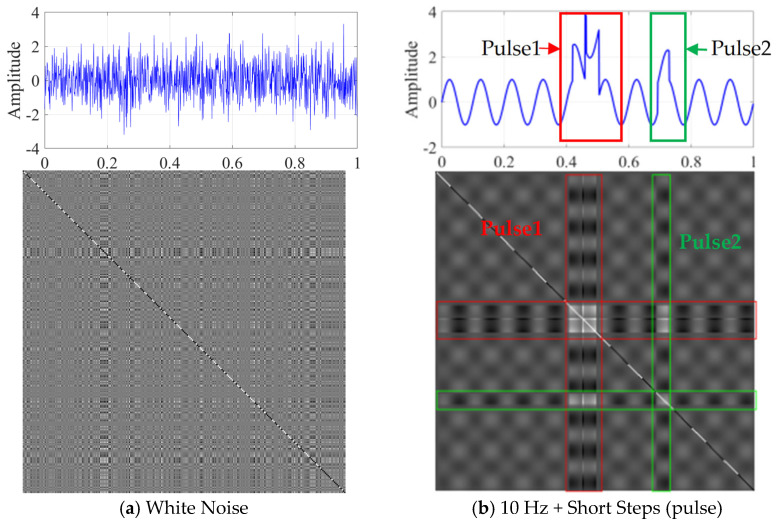
Comparison of GRM generated by different signals.

To provide a clearer understanding of the transformation effect of the GRM on one-dimensional signals, this study investigates various signal types. The four signals utilized in this analysis include: a 10 Hz sine wave, a 20 Hz sine wave, random white noise, and a composite signal that combines a 10 Hz sine wave with a step function. Figure 3 and Figure 4 present a comparative analysis of the GRM images corresponding to these four signal types. As illustrated in Figure 3, the GRM representations of periodic signals exhibit a distinct checkerboard structure, emphasizing their strong periodicity. Moreover, it is observed that as the frequency of the periodic signals increases, the density of the checkerboard pattern also increases correspondingly.

From Figure 4a, it is clear that the random white noise image demonstrates significant randomness, marked by interlaced and scattered black and white dots. Figure 4b illustrates the GRM image of the mixed signal that includes pulses. The pulse sequences (red and green) within the time-domain signal form a row of bright areas at the corresponding horizontal and vertical coordinates in the GRM image. The regions exhibiting alternating bright and dark features correspond to the periodic sine signals within the original time sequence. These results indicate that the GRM method can effectively distinguish the dynamic characteristics of various signal types, revealing the regularity of periodic signals while intuitively reflecting the complexity of random signals and mixed signals. As a result, this approach provides robust support for subsequent feature extraction and fault diagnosis efforts.

To quantitatively evaluate the influence of noise on the GRM method, we calculated the Structural Similarity Index Measure (SSIM) and Peak Signal-to-Noise Ratio (PSNR) separately. These metrics are widely recognized in the academic literature related to image super-resolution [27], which are computed as follows:

(7) $$SSIM=\frac{\left(2\mu_{x}\mu_{y}+C_{1})(2\sigma_{xy}+C_{2}\right)}{\left(\mu_{x}^{2}+\mu_{y}^{2}+C_{1})(\sigma_{x}^{2}+\sigma_{y}^{2}+C_{2}\right)}$$

where μ, $\sigma^{2}$ and $\sigma_{xy}$ represent local means, variances, and covariance, respectively, while $C_{1}$ and $C_{2}$ are small constants to stabilize the division when denominators are close to zero.

(8) $$PSNR=10\cdot \log_{10}(\frac{{\left(\max(S_{ref})\right)}^{2}}{\frac{1}{N}\sum_{i=1}^{N}{\left(S_{rec}(i)-S_{ref}(i)\right)}^{2}})$$

where $S_{ref}$ $S_{rec}$ and N denote the reference, recovered system matrix slice, and the total number of pixels in the slice, respectively.

**Figure 5:**
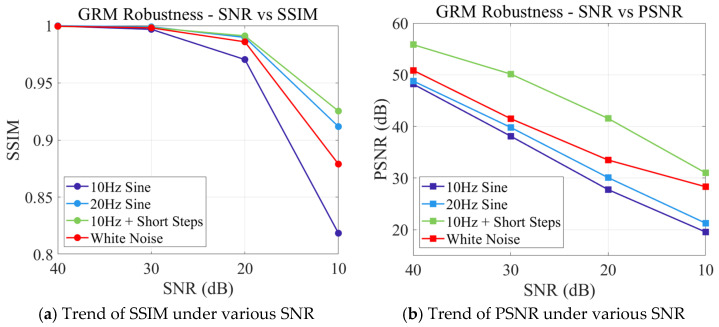
The trend curve of GRM’s sensitivity to noise.

To evaluate the anti-interference capability of the GRM in noisy conditions, this paper investigates its performance indicators using four representative signals at various signal-to-noise ratios (SNR = 40, 30, 20, 10 dB). The results are illustrated in Figure 5. As the SNR decreases, both SSIM and PSNR exhibit a declining trend, suggesting that noise negatively affects the gray-scale structure and overall consistency of the GRM. Notably, even within a high-noise environment with an SNR of 10 dB, the SSIM values for each signal remain between 0.82 and 0.93. This finding highlights the GRM’s ability to effectively preserve relevant structural characteristics, demonstrating strong anti-noise stability.

### 2.3. The Optimized CNN Method

This paper employed and implemented a framework for fault-image identification based on an optimized CNN. Unlike the traditional CNN, the optimized CNN integrates the AdamW optimizer during the training phase [28]. AdamW is a refined version of the standard Adam optimizer. While the Adam optimizer incorporates the weight decay term directly into the gradient computation at each update, AdamW separates the regularization term (weight decay) from the gradient update process. This adjustment allows the weight decay to be applied as an additional term during parameter updates. As a result, the decoupling of weight decay does not hinder the optimization steps, enabling a more effective mitigation of overfitting throughout the training process. The update formula for this approach is as follows:

(9) $$\theta_{t}=\theta_{t-1}-\eta_{t}\frac{\alpha {\hat{m}}_{t}}{\sqrt{{\hat{\nu }}_{t}}+\varepsilon }+\lambda \theta_{t-1}$$

where $\eta_{t}$ is the learning rate and λ is the weight decay coefficient; ε represents a very small constant used to prevent division by zero errors.

**Figure 6:**
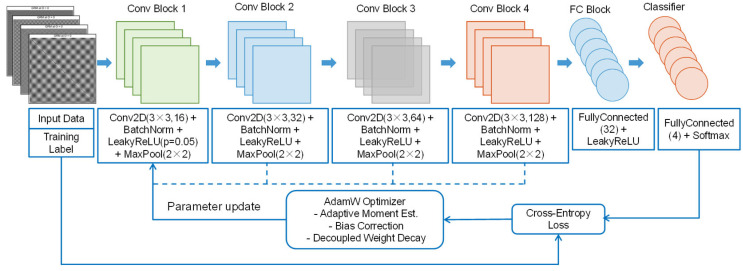
Flowchart of optimized CNN.

The comprehensive methodology employed in the optimized convolutional neural network (CNN) is presented in Figure 6. Initially, images that capture information related to motor faults are collected and used as input data. These images are uniformly scaled to dimensions of 128 × 128 × 3 and appropriately labeled. Subsequently, a refined CNN architecture is established, consisting of four convolutional feature extraction modules (Conv1 through Conv4), a batch normalization layer (BN), and a LeakyReLU activation function. This architecture is designed to enable the hierarchical extraction of multi-scale texture and spatial features. The LeakyReLU activation function is particularly important, as it addresses the “neuron death” phenomenon commonly associated with the ReLU function by retaining non-zero gradients in the negative half-axis. This characteristic enhances the continuity and robustness of feature representation, especially in contexts involving sparse fault features [29]. This network is trained using the cross-entropy loss function with a mini-batch size of 32. During the training phase, the parameters of the CNN are updated through the AdamW optimizer. Metrics related to loss and accuracy are systematically monitored and recorded in real time for thorough evaluation. Once convergence is achieved, the trained and optimized convolutional neural network is employed to analyze the images in the test set for fault classification and prediction.

### 2.4. Overall Process of JMD-GRM-Optimized CNN

**Figure 7:**
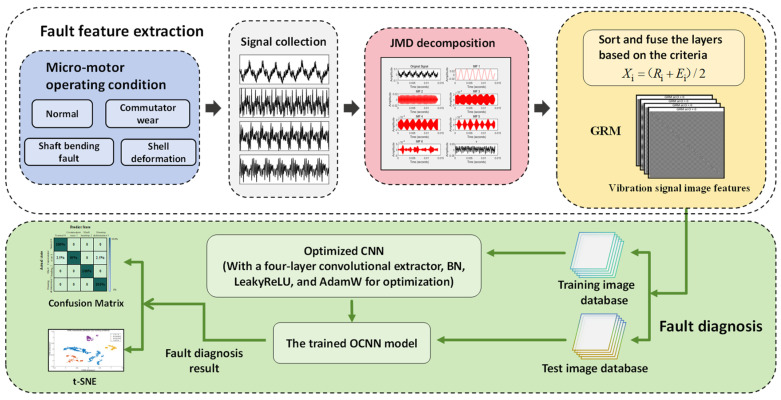
Flowchart of the overall JMD-GRM-optimized CNN.

This paper introduces a systematic methodology for fault diagnosis classification model utilizing JMD-GRM-optimized CNN, as illustrated in Figure 7. The proposed approach comprises the following steps:

1. Sound signal data are gathered from the motor operating under various conditions, namely “normal, commutator wear, shaft bending, and housing deformation”, using an acoustic sensor. This dataset is subjected to JMD, resulting in six layers of modal decomposition. Multiple IMF layers are then selected based on the correlation coefficient-energy model.
2. The chosen IMFs are fused, and GRM visualization processing is applied to the fused signal, leading to the construction of a GRM image library that encapsulates the sound signal data.
3. The GRM image library is divided into a training set and a test set. The training set is employed to train the optimized CNN model, while the test set is used to assess the performance of the trained model.
4. The t-SNE feature visualization results are computed to evaluate the separability of features across different operational states. Following this, the training outcomes, including the confusion matrix, are presented to validate the model’s diagnostic efficacy.

## 3. Experimental Analysis and Verification

**Figure 8:**
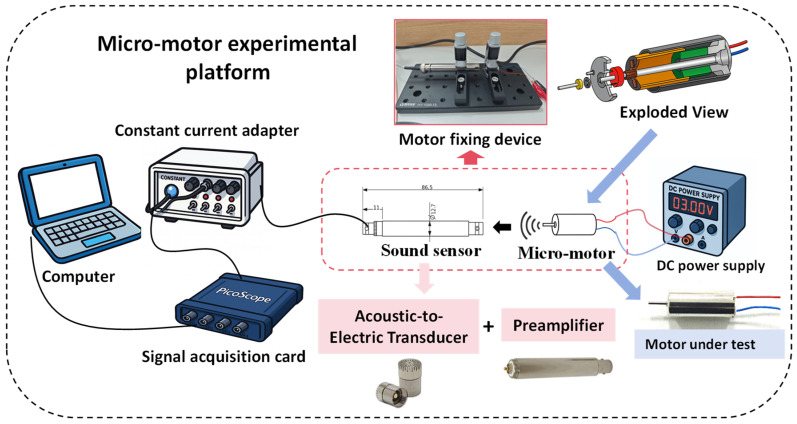
Micro-motor sound signal acquisition platform.

To construct a specialized dataset for micro-motors, this study commenced with the development of a micro-motor testing platform, as illustrated in Figure 8. This platform mainly comprises the motor to be tested (8520 hollow-cup motor, Shenzhen Wanzhida Motor Manufacturing of China, Shenzhen, China), a DC motor drive power supply (DP100 digital control power supply, Guangzhou Xingyi Electronic Technology (Alientek) of China, Guangzhou, China), an acoustic sensor, a signal acquisition card, a computer, and an optical fixation platform. The acoustic sensor operates within a frequency range of 20 Hz to 20 kHz and includes a CT1213 microphone and a CT1201 amplifier (Shanghai Chengke Electronics of China, Shanghai, China). It is powered by a CT5204 constant current adapter (Shanghai Chengke Electronics of China, Shanghai, China) to ensure reliable performance. Signal acquisition is performed using a PicoScope 2406B acquisition card (Pico Technology Company of UK, Cambridgeshire, UK), with a maximum sampling rate of 1 GS/s.

To obtain a representative sample set, multiple repeated experiments were conducted under each specified operating condition. The sampling frequency for each experiment was set at 500 kHz, with a data collection duration of 2 s. During the experimental trials, both the motor under examination and the acoustic sensor were securely affixed to the optical platform using L-shaped optical brackets. The motor was supplied with a 3-volt voltage to ensure operation under no-load conditions, while the acoustic sensor simultaneously collected the sound produced during operation. Signal data were obtained for the motor under four conditions: (1) normal operation, (2) commutator wear, (3) shaft bending, and (4) housing deformation. All subsequent analyses were based on the data set acquired from this experimental framework.

## 4. Fault Diagnosis Analysis of Micro-Motor

### 4.1. Signal Preprocessing

Selecting suitable values for the five critical parameters α, β, b, $\tau_{2}$, K is essential for ensuring both the effectiveness and convergence of the JMD. Therefore, based on parameter range recommendations outlined in reference [15], this study aims to minimize the “sum of sample entropy” as the objective function. Thus, we optimize these five parameters to determine the optimal parameter set. The mathematical representation of the optimization objective function is as follows:

(10) $$\underset{\alpha ,\beta ,b,\tau_{2},K}{min}F(\alpha ,\beta ,b,\tau_{2},K)=\sum_{i=1}^{K}SE_{i}(m,r,N)$$

Here $SE_{i}(m,r,N)$ represents the sample entropy of the *i*-th intrinsic mode component (IMF), and K is the number of decomposition layers. By minimizing the objective function F, an optimal set of parameters $\left(\alpha ,\beta ,b,\tau_{2},K\right)$ can be obtained to achieve a better JMD effect. By solving the optimization function, several parameters were finally set as: $\alpha =10^{4}$, β=0.35, b=0.32, $\tau_{2}=3$, K=6. Under this set of parameters, JMD yields better results.

The selection of an appropriate number of decomposition variables significantly influences the representation of input features in GRM images. Therefore, a comprehensive analysis of the IMFs is necessary to determine their relative importance. The significance of each IMF layer is assessed using two primary indicators: the correlation between the decomposed IMF components and the original signal, and their corresponding energy. A correlation coefficient-energy model is employed to select the IMFs, with the top 50% ranked IMFs identified as the final layers for further analysis. After this selection, the fused signal comprising IMF1, IMF2, and IMF3 is used as input to the GRM channel, which enables more accurate evaluation of fault states and classifications. The application of GRM to enhance signal features further facilitates clearer differentiation among various fault characteristics.

### 4.2. Analysis of Fault Diagnosis Results of Micro-Motors

**Figure 9:**
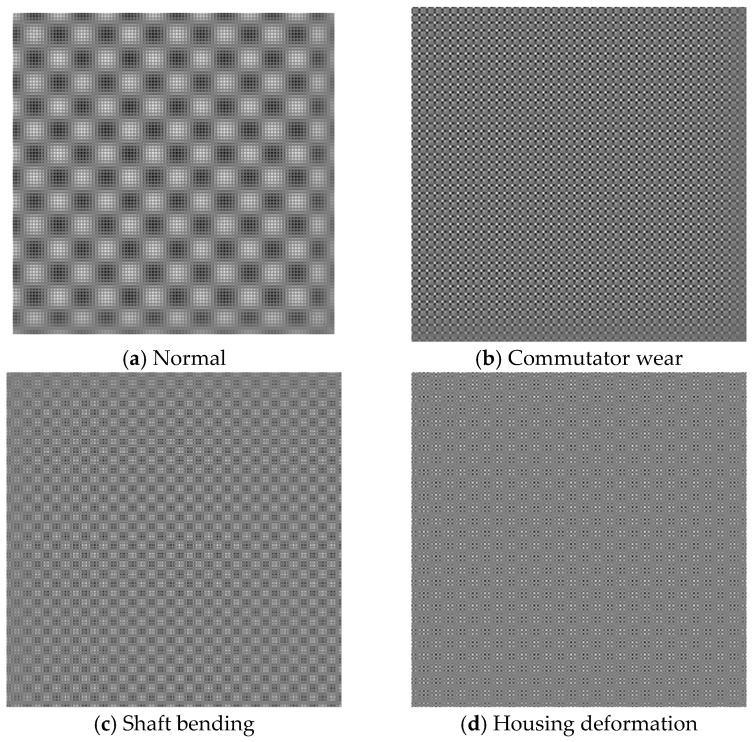
GRM diagrams under different operating conditions.

A set of GRM diagrams under four different operating conditions is shown in Figure 9. A symmetrical comparison of the images along the diagonal reveals significant variances among the various operating conditions, indicating differing degrees of distinction in the observed fault characteristics.

In this paper, the optimized CNN method is used for machine learning and fault classification. The JPG images in the sample dataset are organized into four distinct folders, each assigned a specific label: “normal” as label 0, “commutator wear” as label 1, “shaft bending fault” as label 2, and “housing deformation” as label 3. The dataset comprises 750 training images, including 425 images representing normal working conditions and 105 images for each fault condition. The test dataset consists of 210 images, with 90 depicting normal conditions and 40 representing each type of fault. To ensure effective input of the JPG images into the optimized CNN for both training and evaluation, preprocessing operations are performed to standardize the dimensions of the images, ensuring uniformity across all inputs.

Six models were evaluated for classifying motor faults: JMD-GRM-optimized CNN, VMD-GRM-optimized CNN, JMD-RP-optimized CNN (RP, Recurrence Plot), JMD-RPM-optimized CNN (RPM, Relative Position Matrix), JMD-GRM-CNN, and JMD-GRM-SVM. The fault identification results of the six models are presented in Table 1. The results indicate that the JMD-GRM-optimized CNN model achieved superior performance, with an accuracy rate of 99.0476%. Additionally, this model exhibited a standard deviation of only ±0.3% across five independent runs, suggesting minimal performance fluctuation and demonstrating the robustness and stability of the proposed approach.

**Figure 10:**
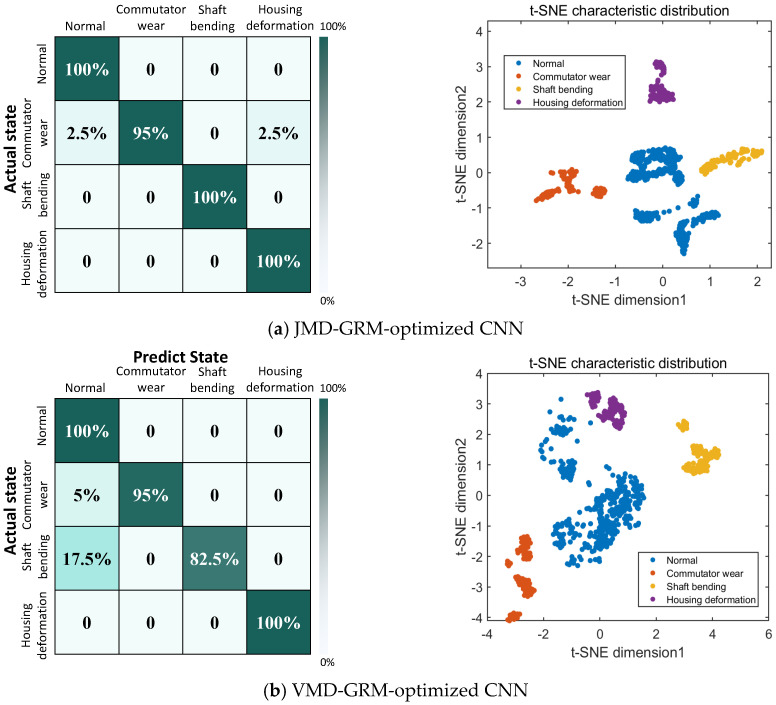

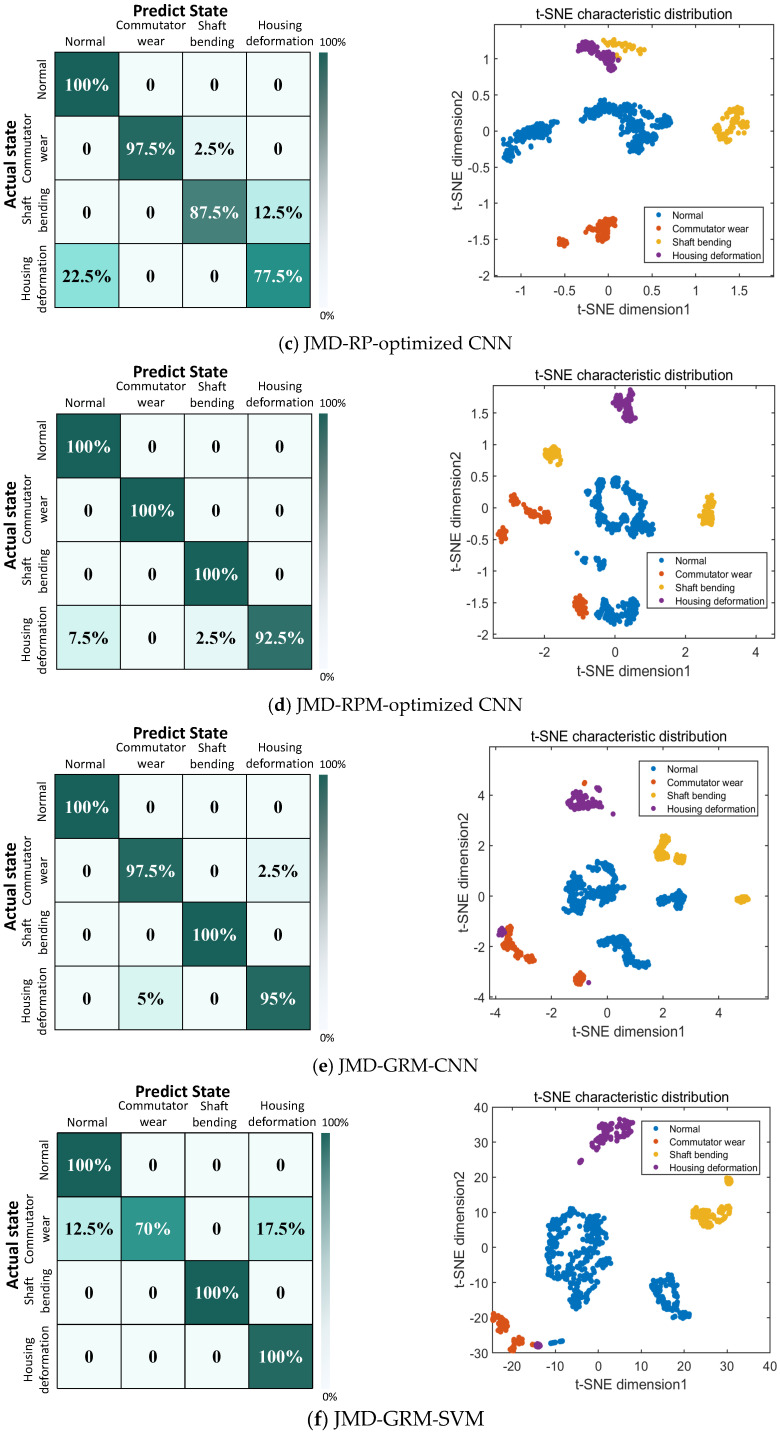
Confusion matrices and t-SNE under different models of micro-motor dataset.

Figure 10 presents the confusion matrices and t-SNE (t-distributed Stochastic Neighbor Embedding) visualizations of six distinct models. In the t-SNE plots, the sample categories are represented as follows: normal (blue), commutator wear (orange), shaft bending (yellow), and housing deformation (purple). These t-SNE representations are derived from the output of the penultimate layer of each model. These figures demonstrate that the samples generated by the method proposed in this study exhibit the highest level of concentration, thereby underscoring the efficacy of the approach.

The analysis of the confusion matrix reveals that the VMD-GRM-optimized CNN model effectively identifies normal conditions and housing deformation faults. However, it encounters difficulties in accurately classifying commutator wear faults, resulting in some misclassifications, while also showing relatively low recognition accuracy for shaft bending faults. The JMD-RP-optimized CNN model is characterized by significant misjudgments in fault identification, particularly with housing deformation faults, where the rate of misclassification is notably high. Although the JMD-RPM-optimized CNN model demonstrates high accuracy in recognizing all other fault states, it similarly experiences challenges with housing deformation faults. In contrast, the JMD-GRM-CNN model successfully distinguishes normal states and shaft bending faults, though it struggles to differentiate between commutator wear and housing deformation faults. Furthermore, The JMD-GRM-SVM model accurately identifies all operating conditions except commutator wear. However, it tends to confuse commutator wear and normal operating conditions with shell deformation faults. In comparison to the previously discussed models, the JMD-GRM optimized CNN model exhibits superior predictive performance.

### 4.3. Analysis of Results from the CWRU Bearing Dataset of Western Reserve University

To further evaluate the effectiveness and robustness of the proposed method in this paper, we conducted a performance validation using the publicly available bearing dataset from Case Western Reserve University (CWRU). The analysis was carried out under no-load conditions at a rotational speed of 1797 revolutions per minute, with a fault diameter of 0.007 inches. We examined four distinct scenarios: normal operation, inner race fault, ball fault, and outer race fault, all maintaining the same fault diameter of 0.007 inches. Following this, we performed classification accuracy calculations for each of these fault conditions, enabling a thorough assessment of the model’s performance.

**Figure 11:**
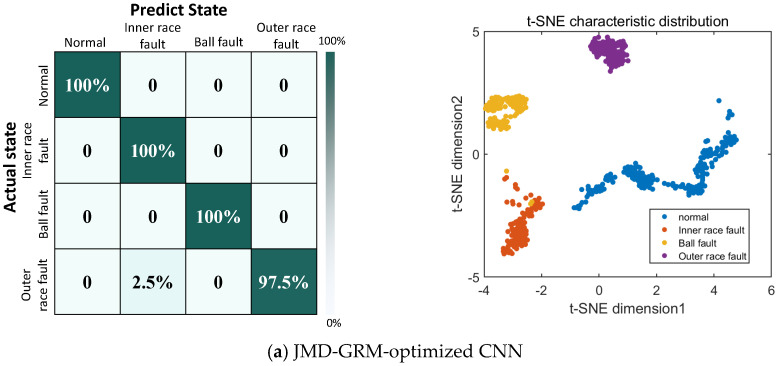

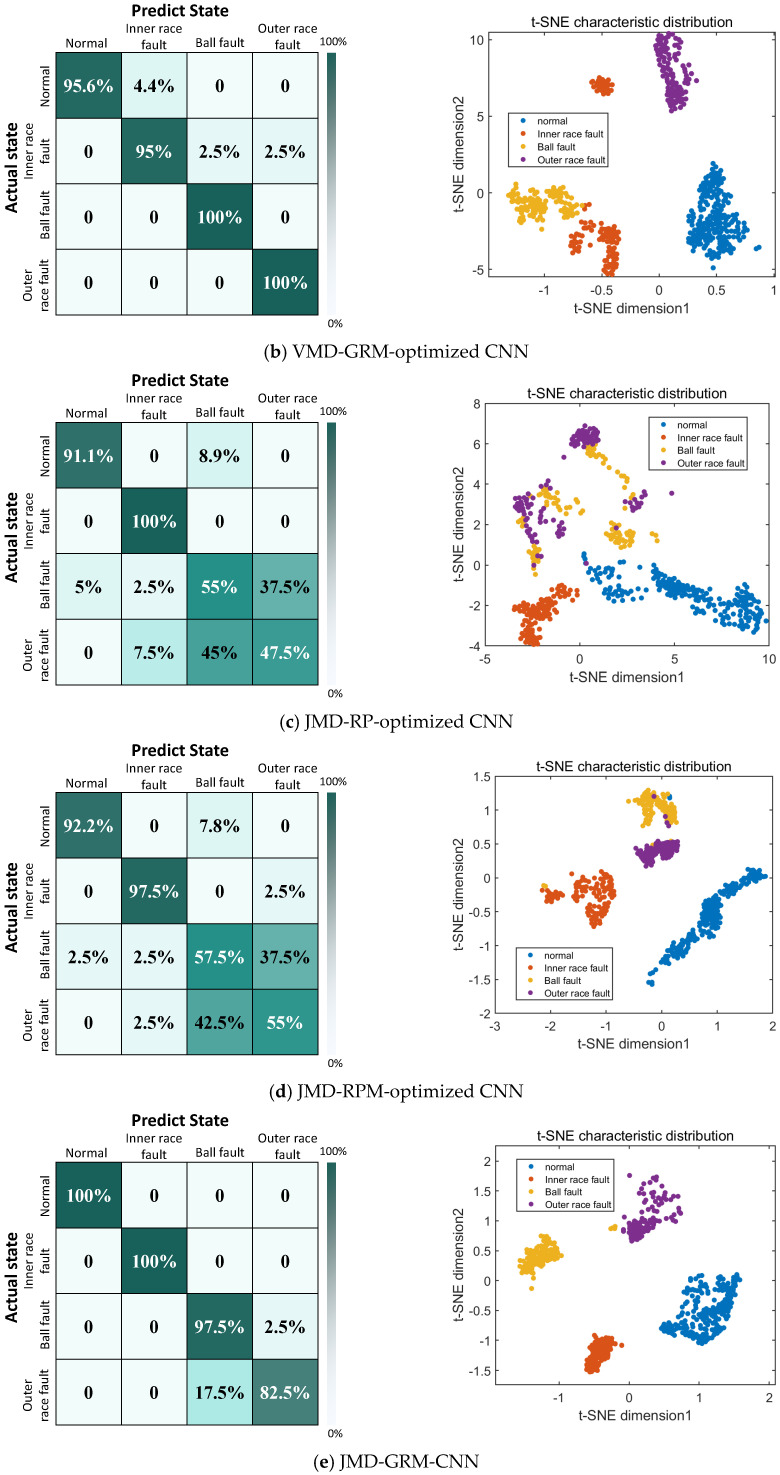

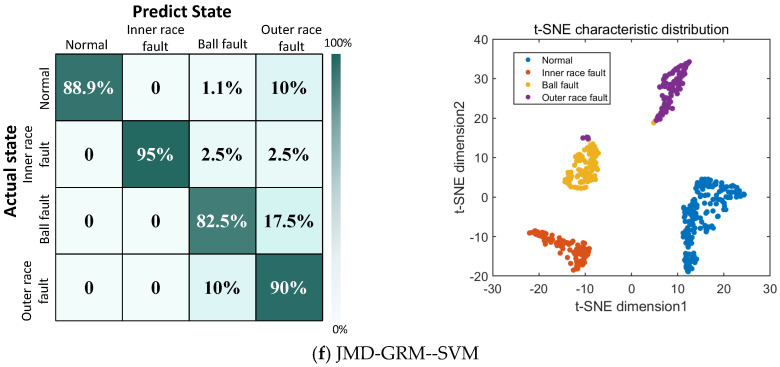
Confusion matrices and t-SNE under different models of CWRU dataset.

Initially, the JMD parameter group was configured to: $\alpha =10^{5}$, β=0.86, b=0.35, $\tau_{2}=1.1$, K=6, utilizing the particle swarm optimization algorithm. The final classification accuracy rates for each model are detailed in Table 2. Furthermore, the confusion matrices and t-SNE plots that illustrate the classification outcomes for the six models are presented in Figure 11. Both Table 2 and Figure 11 provide evidence that the JMD-GRM-optimized CNN model exhibits superior performance, achieving a classification accuracy rate of 99.4286%, which surpasses that of the other models evaluated.

## 5. Conclusions

This study introduces a novel methodology for diagnosing faults in micro-motors, utilizing an optimized CNN in combination with a JMD-GRM. This approach replaces the conventional dependence on high-precision vibration sensors with low-cost acoustic signals, enabling accurate identification of various micro-motor faults. Beyond cost reduction, the method addresses challenges in feature extraction from weak acoustic signals, facilitating efficient detection of multiple fault types.

(1) The JMD method effectively addresses the challenge of signal aliasing, which is a common concern in traditional signal processing techniques. Through the decomposition and reconstruction of non-stationary signals, this method demonstrates significant advantages in the analysis of jump signals and is well-suited for separating the characteristic components of motor fault signals.
(2) The GRM method was introduced to visualize the fused signal effectively, and its susceptibility to noise was systematically evaluated. The resulting GRM images served as inputs for the optimized CNN fault classification model, which improved the efficiency and accuracy of fault classification.
(3) A comprehensive multi-fault classification test system was developed to assess the effectiveness of the proposed methodology. Experimental results indicate that the AdamW optimizer substantially improves the performance of the traditional CNN model, leading to higher classification accuracy and faster training convergence, particularly in motor fault diagnosis. Six distinct fault diagnosis models were implemented, and the proposed approach was evaluated using both a self-constructed dataset and the publicly available CWRU dataset. The method achieved fault classification accuracy rates of 99.0476% and 99.4286%, respectively, surpassing the performance of the other methods.

## Figures and Tables

**Table 1:** Comparison of prediction results of micro-motor dataset.

Method	JMD-GRM-Optimized CNN	VMD-GRM-Optimized CNN	JMD-RP-Optimized CNN	JMD-RPM-Optimized CNN	JMD-GRM-CNN	JMD-GRM-SVM
Mean Average Accuracy	99.0476%	95.7143%	92.4762%	98.1905%	98.5714%	94.2857%
Standard Deviation (Std)	±0.30%	0	±2.14%	±0.19%	±0.30%	0

**Table 2:** Comparison of prediction results of CWRU bearing dataset.

Method	JMD-GRM-Optimized CNN	VMD-GRM-Optimized CNN	JMD-RP-Optimized CNN	JMD-RPM-Optimized CNN	JMD-GRM-CNN	JMD-GRM-SVM
Mean Average Accuracy	99.4286%	97.3333%	77.9048%	79.7143%	96.1905%	89.0476%
Standard Deviation (Std)	±0.36%	±0.23%	±3.52%	±3.36%	±0.52%	0

## Data Availability

The original contributions presented in this study are included in the article. Further inquiries can be directed to the corresponding author.
